# Coronary embolization from aortic valve fibroelastoma

**DOI:** 10.1002/ccr3.2954

**Published:** 2020-05-20

**Authors:** Henry O. Savage, Alberto Albanese, Vincenzo Caruso, Swamy Gedela, Jason Dungu

**Affiliations:** ^1^ Department of Cardiology Medicine and Cardiothoracic Surgery Basildon and Thurrock University Hospital Basildon UK; ^2^ Department of Cardiac Surgery St Thomas’ Hospital London UK

**Keywords:** aortic valve, cardiac imaging, papillary fibroelastoma

## Abstract

Papillary fibroelastomas have a range of clinical presentations. The surgical removal of these tumors should always be considered as best alternative to a conservative approach.

## Graphical abstract

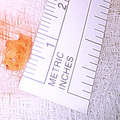

## 1 INTRODUCTION

We present the case of a 39‐year‐old female patient with sudden onset of chest pain and diagnosis of acute myocardial ischemia. A transesophageal echocardiogram showed a large mobile mass attached to the aortic side of the left coronary cusp of the aortic valve.

Papillary Fibroelastomas (PFE) are rare tumors of the heart. They are the third most common cardiac tumors after myxomas and lipomas, accounting for approximately 10% of all benign primary cardiac tumors.^1^ They are typically incidental findings and usually found on cardiac valves, but they can be found on the papillary muscles, chordae tendineae, the ventricular septum, or the endocardial surface.^2^ Clinical manifestation of a PFE varies from asymptomatic to severe embolic sequelae.^3^

## 2 CASE REPORT

A previously fit and well 39‐year‐old lady presented with central crushing chest pain, radiating to her left arm. Her initial 12‐lead electrocardiogram (ECG) showed sinus rhythm with transient ST‐segment elevation in the anterior chest leads, which settled as fixed anterolateral T‐wave inversion on subsequent ECGs. This, along with a significant cardiac enzyme rise, confirmed a diagnosis of an aborted anterior ST‐elevation myocardial infarction.

A bedside transthoracic echocardiogram (TTE) demonstrated a mobile mass on a structurally normal aortic valve. There was also noted an acutely impaired left ventricle (LV) with an ejection fraction (EF) of 35%‐40% and regional wall motion abnormalities involving the anterior wall and apex.

**Figure 1:**
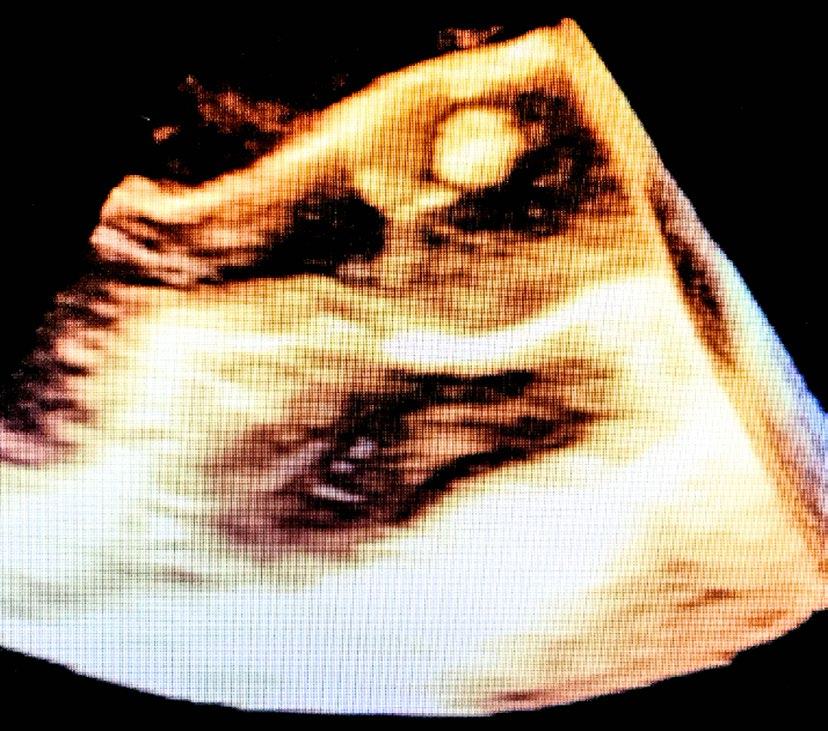
Full‐volume 3D TOE demonstrating pedunculated fibroelastoma attached to left coronary cusp of aortic valve

A transesophageal echocardiogram (TOE) revealed a highly mobile pedunculated mass attached to the aortic side of the left coronary cusp of the aortic valve, measuring 1 cm^2^ (Figure 1). There were no other structural valve abnormalities noted.

Due to the proximity of the mass to the coronary ostia, a computed tomography coronary angiogram (CTCA) was performed which revealed an occluded first diagonal artery (D1). The remainder of both coronary systems was smooth and unobstructed.

**Figure 2:**
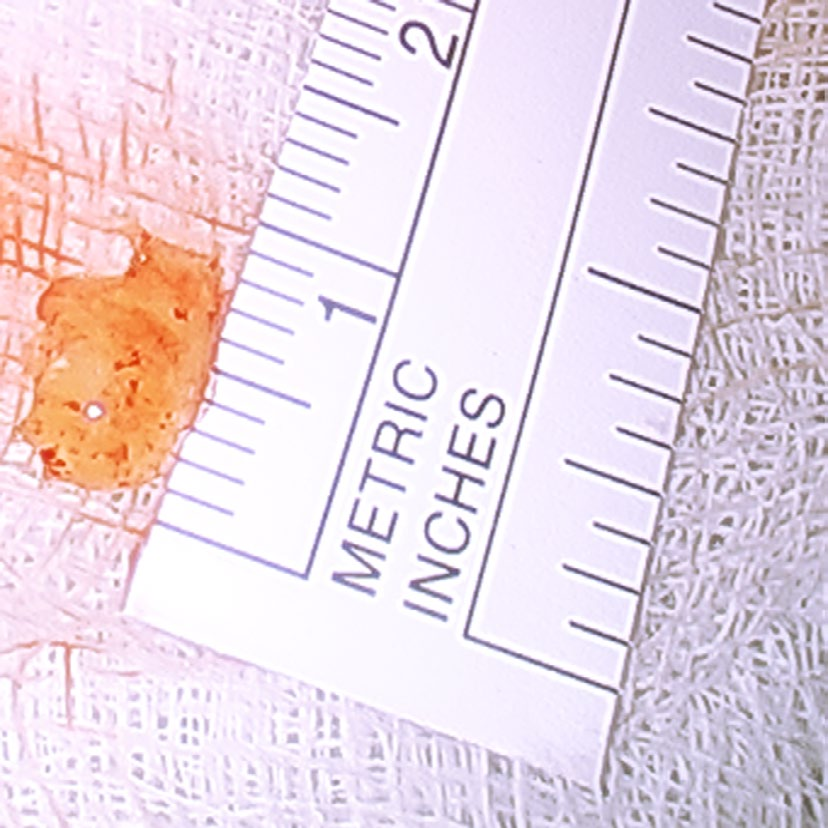
Gross specimen of the fibroelastoma

A repeat TTE confirmed recovery of myocardial function with an EF measuring 50%. Given the improvement in LV function, a second CTCA was arranged to guide the decision for surgical coronary revascularization. This revealed that the previously occluded D1 was now unobstructed with normal flow. Intraoperatively, a single phylliform mass was observed lying on the aortic side of the left coronary cusp. It had multiple papillary fronds connected to the leaflet by a thin pedicle. The mass appeared friable with a diameter of around one centimeter (Figure 2). No other abnormalities were observed on the aortic valve or in the ventricular outflow tract.

A valve‐sparing approach was attempted and the mass was completely removed at its pedicle, with no aortic leaflet deficit or macroscopic changes in its morphology. Finally, the aortic valve was tested for competency using a hydrodynamic saline tester, which demonstrated no evidence of aortic regurgitation, good mobility of all the leaflets, and a satisfactory coaptation area.

A histopathology examination confirmed the diagnosis of PFE comprising of papillary fronds featuring a central core of dense connective tissue surrounded by a layer of loose connective tissue and covered by a layer of plump endothelial cells. There was also noted focal calcification but no cytological atypia.

## 3 DISCUSSION

Primary cardiac tumors have a prevalence of 0.02%. About 75% of these tumors can be classified as benign and 25% as malignant. Papillary fibroelastomas are less common than myxomas. The cardiac valves are predominantly involved; the aortic valve is often affected followed by the mitral valve, and concomitant valvular dysfunction occurs in less than half of cases.^4^ The most common clinical presentation, in symptomatic cases, is embolism, presenting as transient ischemic attacks or stroke, and the female sex had a greater incidence. The mean size of tumors ranges from 2 to 70 mm.^5^

There is no consensus about the etiology of PFE, and therefore, it is common to classify this entity as a “tumor‐like lesion” rather than a genuine neoplasm. In the 4th edition of the WHO classification of tumors of the lung, pleura, thymus and heart, it is noted that there is no histological or molecular proof to support a true neoplastic origin of papillary fibroelastomas.^6^ In this context, it has been hypothesized that the continuing turbulent blood flow in the heart and the consequent hemodynamic trauma of the endothelium contribute to the development of papillary fibroelastomas.^7^ Moreover, PFE are frequently difficult to differentiate from Lambl's excrescences, in terms of microscopic features, clinical sequalae, and cardiac localization, even though Lambl's excrescences are usually smaller and broader‐based.^8^

The use of noninvasive imaging modalities such as TOE and computed tomography is invaluable in characterizing these lesions and to determine the status of the coronary arteries.

Depending on the size and mobility of the PFE, patients may present with acute coronary syndromes, as a result of dynamic coronary ostia occlusion or embolization. This is not an unusual postmortem finding, in patients who present with sudden cardiac death. However, acute coronary syndromes occur less frequently compared to cerebrovascular events or as an incidental finding.

Our literature search using the terms “papillary fibroelastoma” and “coronary artery” identified only 26 such publications, written in English language within an interval of 10 years to date, which correlate PFE with coronary artery presentations. There are no clear indications for surgery in asymptomatic patients with small nonmobile tumors, and a conservative approach with serial imaging seems to be the general consensus. In symptomatic patients, however, valve‐sparing surgical excision of the tumor is the goal of management. A comprehensive coronary artery assessment is recommended prior to surgery to determine the need for concomitant surgical myocardial revascularization.

This report confirmed a dynamic coronary artery occlusion from potential embolized components of a PFE, in a patient who presented with an acute ST‐elevation myocardial infarct. The territories of myocardial dysfunction initially noted at presentation suggest that there was migration of embolus through the left main‐left anterior descending artery before settling in the first diagonal, as noted on the first CTCA. The dynamic nature of this embolus is confirmed by the subsequent CTCA, which revealed resolution of occlusion in D1. This change demonstrates the high embolization potential of PFE even in erstwhile small‐sized tumors and correlates with autopsy findings in patients who present with sudden cardiac death.

We suggest that surgical treatment should always be a considered option in patients in whom this tumor is found even when they appear to be small. Where possible, surgical treatment should focus on complete tumor removal with preservation of the anatomical structure of the affected valve.

## CONFLICT OF INTEREST

None declared.

## AUTHOR CONTRIBUTIONS

Dr Savage: made substantial contributions to conception and design and acquisition of data, has been involved in revising the manuscript critically for important intellectual content, and has given final approval of the version to be published. Dr Albanese: made substantial contributions to conception and design and acquisition of data and has given final approval of the version to be published. Dr Caruso: made substantial contributions to conception and design and acquisition of data; has been involved in drafting and revising the manuscript critically for important intellectual content, and has given final approval of the version to be published. Dr Gedela: made substantial contributions to conception and design and acquisition of data and has given final approval of the version to be published. Dr Dungu: made substantial contributions to conception and design and acquisition of data and has given final approval of the version to be published. All the authors agreed to be accountable for all aspects of the work in ensuring that questions related to the accuracy or integrity of any part of the work are appropriately investigated and resolved.

## ETHICAL STATEMENT AND THE CONSENT STATEMENT

Appropriate consent has been obtained, prior to submission, for the publication of imagines and data.
